# Supplementary material for: CUT&Tag applied to zebrafish adult tail fins reveals a return of embryonic H3K4me3 patterns during regeneration
Source: Epigenetics Chromatin. 2024 Jul 20;17:22. doi: 10.1186/s13072-024-00547-5 (PMC11264793; doi:10.1186/s13072-024-00547-5)

Supplementary Figure 1

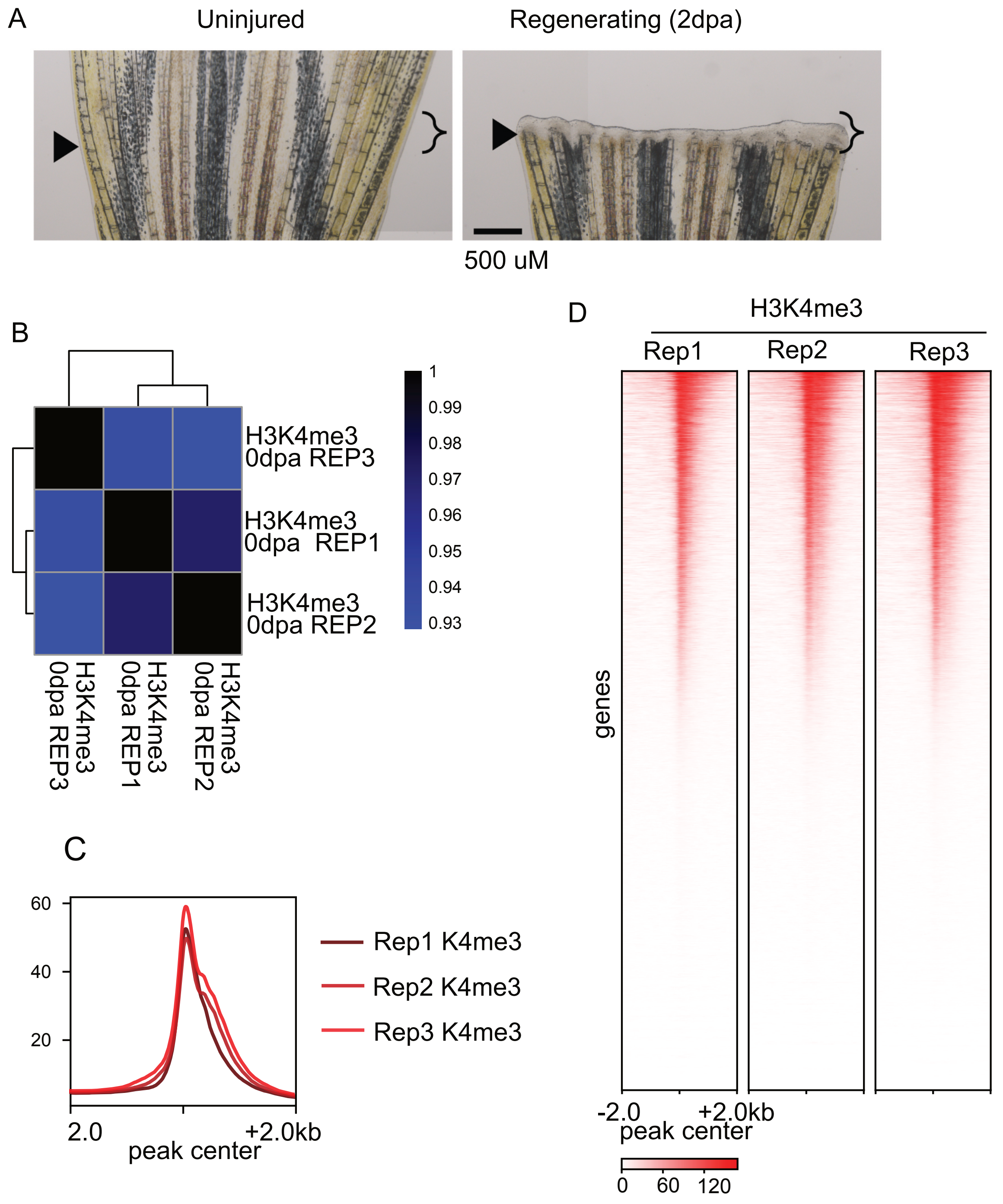

Supplementary Figure 2

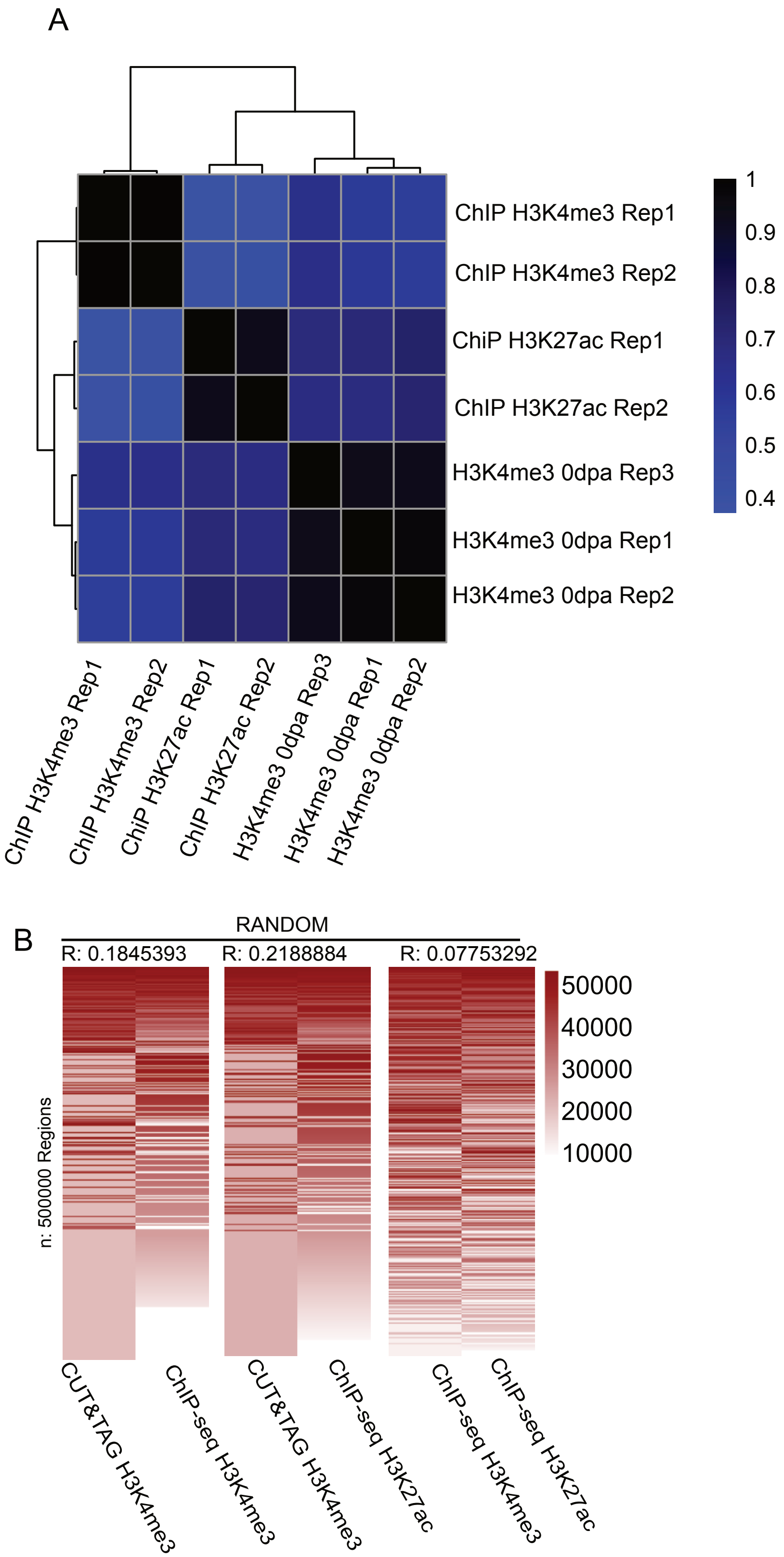

Supplementary Figure 3

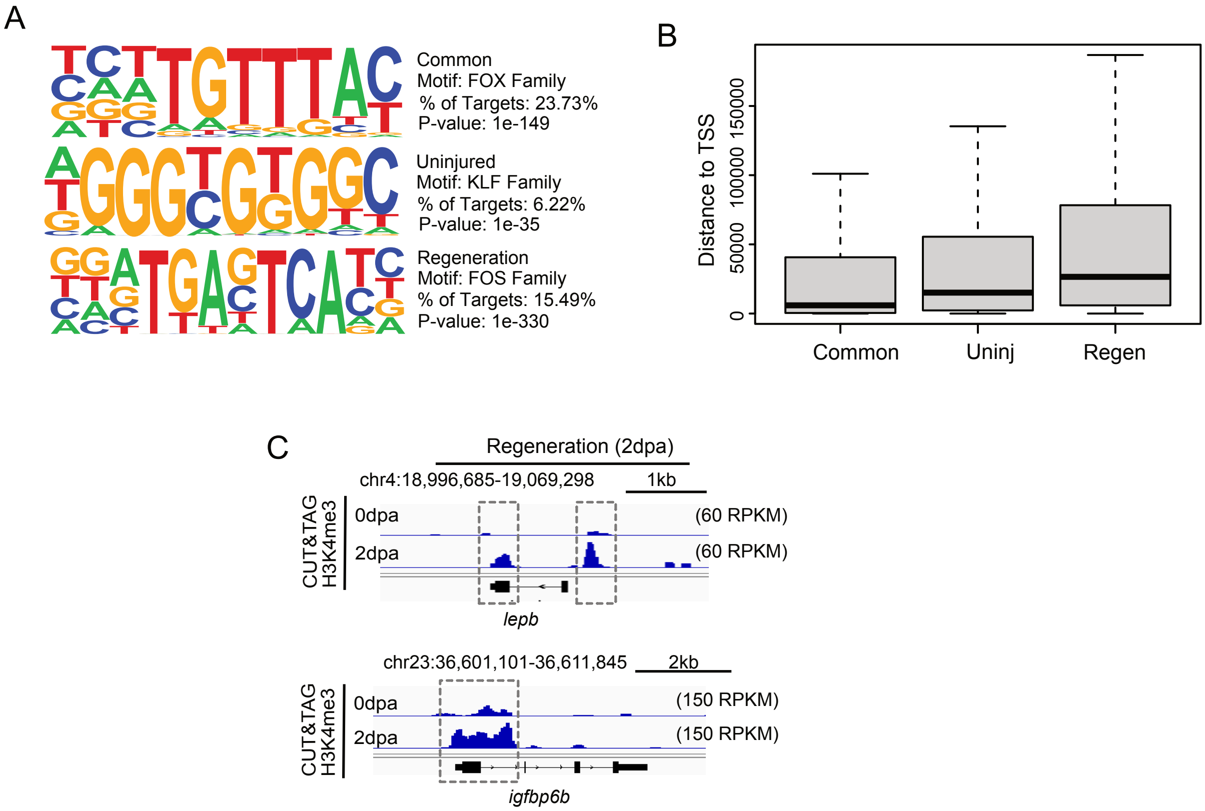

Supplementary Figure 4

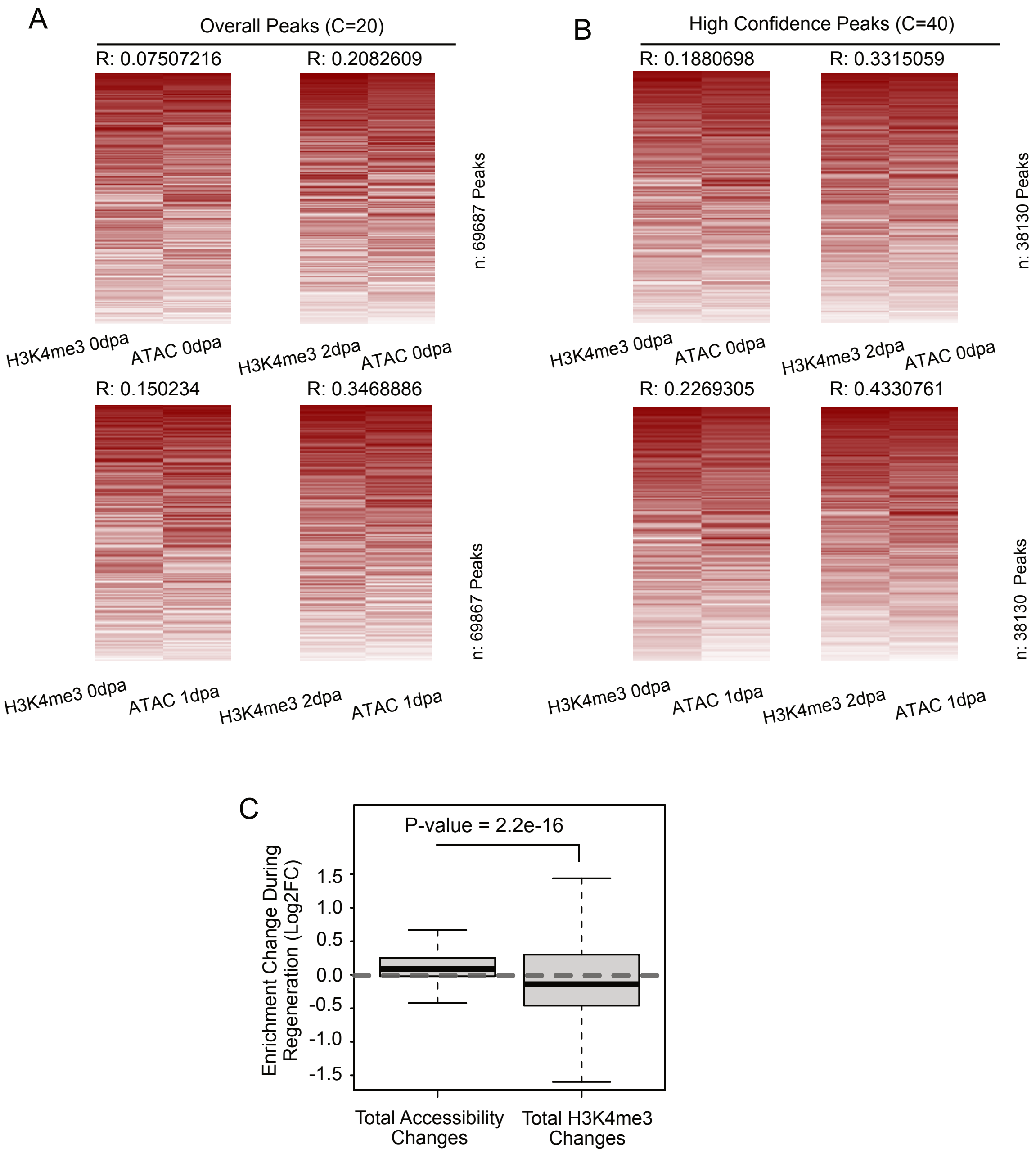

Supplementary Figure 5.

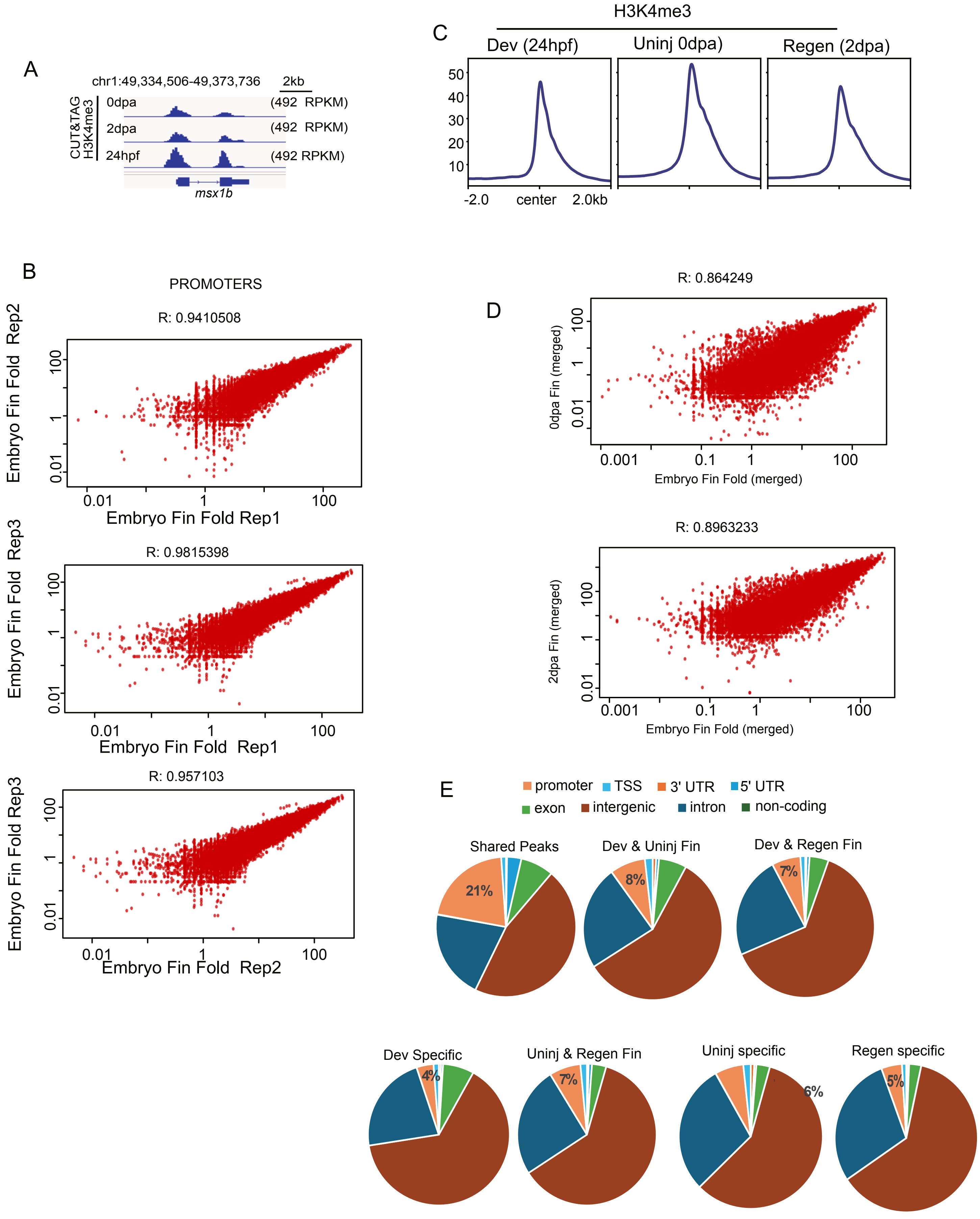

Supplementary Figure 6.

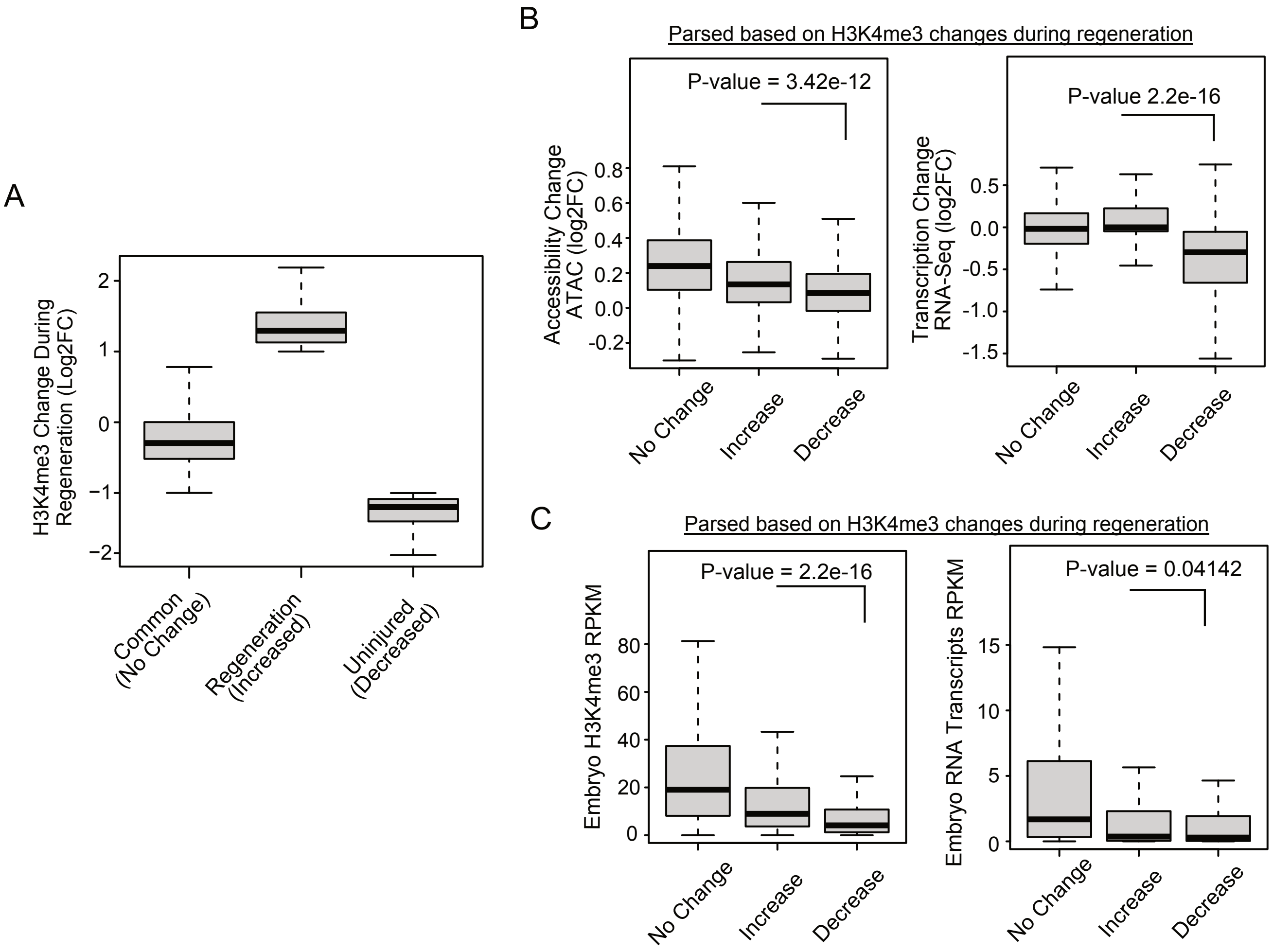

Supplement: Supplementary file 1 — Supplementary Material 1: Supplementary Figure 1. (a) Brightfield images of unamputated zebrafish caudal fin and 2 dpa fin. (b) Pearson correlation values are plotted as a heatmap in pair-wise matrix format comparing individual H3K4me3 Uninjured (0 dpa) CUT&Tag replicates. (c) Profile plots of three individual H3K4me3 CUT&TAG replicates at the promoter genes with H3K4me3 signals as detected by CUT&TAG in zebrafish fins. (d) Heat maps of individual 0 Uninjured (0 dpa) replicate data for H3K4me3 enrichment (RPKM) from CUT&Tag at the TSS of annotated genes. Supplementary Figure 2. (a) Pearson correlation values are plotted as a heatmap in pair-wise matrix format comparing CUT&Tag for H3K4me3 with ChIP-Seq from H3K4me3 and H3K27ac. (b) Rank normalized heatmap demonstrating low correlation between CUT&Tag and ChIP-Seq when assessed over random non-enriched genomic regions. Supplementary Figure 3. (a) Enriched transcription factor binding motifs for region with H3K4me3 enrichment classified as Common/ Uninjured (0 dpa)/Regeneration (2 dpa) in zebrafish fins. (b) Box plots displaying the average distance to gene transcription start sites for each set of peaks in Common, Uninjured (0dpa), and Regeneration (2 dpa) fin categories. (c) Genome browser view showing enrichment of H3K4me3 at putative regulatory elements for selected genes. Supplementary Figure 4. (a) Rank normalized heatmap demonstrating low correlation between CUT&Tag and ATAC-Seq datasets generated from regenerating zebrafish fin tissues. Pearson correlation values are displayed. (b) Rank normalized heatmap with high confidence peaks demonstrating higher correlation between CUT&Tag and ATAC-Seq datasets generated from regenerating zebrafish fin tissues. Pearson correlation values are displayed. (c) Boxplots of enrichment change during regeneration for changes in chromatin accessibility and H3K4me3. Supplementary Figure 5. (a) Genome browser view showing enrichment of H3K4me3 at the msxb1 gene. (b) Scatter plots [file 13072_2024_547_MOESM1_ESM.pdf]
