# Supplementary material for: CUT&Tag applied to zebrafish adult tail fins reveals a return of embryonic H3K4me3 patterns during regeneration
Source: Epigenetics Chromatin. 2024 Jul 20;17:22. doi: 10.1186/s13072-024-00547-5 (PMC11264793; doi:10.1186/s13072-024-00547-5)
Supplement: Supplementary file 2 — Supplementary Material 2. [file 13072_2024_547_MOESM2_ESM.pdf]

Table of Contents

Dissociation to get nuclei for CUT&Tag 3

Optimizing Zebrafish Dissociation & CUT&Tag 3

General Notes 3

Enzyme Assessment 3

Adult Caudal Fin Dissociation 7

Buffers 7

Procedure 7

Embryo Fin Fold Dissociation 10

Buffers 10

Procedure 10

Mating 10

Dechorionating 11

Cutting Fin Fold 12

Fin Fold Dissociation 15

Protocol for CUT&Tag 17

Buffers 17

Antibodies 19

Procedure 19

Nuclei fixation and binding nuclei to activated beads: 19

ConA bead activation: 20

Primary and secondary antibodies binding: 21

Binding of pAG-Tn5: 22

Targeted chromatin tagmentation: 22
Quality control: 24
Library preparation and cleanup: 25
DNA cleanup using AMPure XP beads 25
Antibody Assessment 26
Library Primer Information 28

Dissociation to get nuclei for CUT&Tag

Optimizing Zebrafish Dissociation & CUT&Tag

Cell dissociation from the tissue is the most crucial step for the CUT&Tag experiment. Below you will find tables and graphs to evaluate the dissociation protocol that we tested.

GENERAL NOTES

- Resuspend cells and nuclei **very** gently. Any time the vortex is used, it should be set to the lowest setting. When pipetting up and down to resuspend cell pellets, eject the cells slowly from the pipette tip. Ejecting cells (slowly) in a circular pattern near the bottom of the tube may also help to resuspend cells without using more force.

- Using > 60k nuclei provides more **consistent** results. Using lower nuclei numbers works, but the result may be inconsistent. Using over 100k cells is preferred, as less cells may be lost during nuclear extraction and fixation during the CUT&Tag procedure (See CUT&Tag section).

- If using uninjured/2 days post-amputation (dpa) whole fins, use 5 fins / 2 antibodies (abs; control IgG and experimental ab) as a minimum. Any lower than 5 fins, there is significant cell loss during dissociation (even with BSA-coated tubes).

- Changing centrifugation speed may be applied based on the desired cell types. Using transgenic lines to FACS sort cells, we recommend testing a range of different centrifugation speeds. We noticed that 300xg pellets mainly large and heavy cells, and 600xg pellets small, light, and large cells. We also found no improved results obtained from centrifuging cells at speeds higher than 600xg.

ENZYME ASSESSMENT

Previous papers employed distinct tissue dissociation protocols (**Table 1**). It may be beneficial to further optimize a dissociation buffer based on desired tissue/cell types. **Table 2** lists the

optimization methods that we tested.

Table1: Review of enzymatic digestion buffers.

Reference	Enzyme 1	Enzyme 2	Tissue Type
**Sander et al., 2013**	Collagenase Type II: 5mg/mL	Collagenase Type IV: 5mg/mL	Adult zebrafish hearts
**Mouloungui et al., 2018**	Collagenase Type I: 200IU/mL		Human preantral follicles
	Collagenase NB6: 200IU/mL		
	Liberase DH: 0.28U/mL		
**Pimpalwar et al., 2020**	Liberase DH: unspecified		Fresh mouse heart
**Li et al., 2021**	Collagenase Type I: 1mg/mL	Dispase II: 2U/mL	Mouse hind-limb muscles
**Akdogan-Ozdilek et al., 2021**	Collagenase P: 2mg/mL	Trypsin: 0.25%	Zebrafish embryo

Table 1: Sander et al. (2013) used Collagenase type II and IV for zebrafish heart dissociation optimized for cardiomyocyte culturing. Li et al. (2021) used Collagenase type I and Dispase for mouse hind-limb muscle dissociation. Mouloungui et al. (2018) methodically tested a dissociation technique of human preantral follicles using three different proteases. They tested 200IU/mL of collagenase type I, 200IU/mL of collagenase NB6, and of 0.28U/mL of Liberase DH. They found no difference in number of cells isolated or survival rate between the three enzymes. Pimpalwar et al. (2020) also tested different enzyme-based dissociation protocols of human and mouse heart. They found a greater number of valid cells per µL using Liberase digestion on freshly dissected mouse hearts. Concentration or type of Liberase used was not mentioned in the methods section. Liberase DH has collagenase I, II and Dispase.

Table 2: Optimizing dissociation for Zebrafish tissues.

Buffer	Collagenase type IV final conc.	Liberase DH final conc.	Temperature	Tissue Type	Cell survival
**Perfusion Buffer***	5 mg/mL	0.26 U/mL	28°C	Fin	Un
**Perfusion Buffer***	5 mg/mL	0.26 U/mL	37°C	Uninj. fin	A
**Perfusion Buffer***	5 mg/mL	0.26 U/mL	33°C	Uninj. fin	A
**Perfusion Buffer***	5 mg/mL	0.26 U/mL	37°C	2 dpa fin	A
**Perfusion Buffer***	5 mg/mL	0.26 U/mL	33°C	2 dpa fin	A
**Perfusion Buffer***	5 mg/mL	0.26 U/mL	35°C	3 dpi Heart	A
**HBSS**	5 mg/mL	0.26 U/mL	35°C	3 dpi Heart	A
**HBSS**	5 mg/mL	0.52 U/mL	33°C	Fin	Un
**HBSS**	5 mg/mL	0	33°C	Fin	Un
**HBSS**	0	0.52 U/mL	33°C	Fin	Un
**HBSS**	5 mg/mL	0.26 U/mL	35°C	Fin	A
**HBSS**	5 mg/mL	0.26 U/mL	35°C	2 dpa fin	A
**HBSS**	5 mg/mL	0.26 U/mL	35°C	Heart	A

Table 2: We began testing zebrafish fin dissociation by determining if fins should be dissociated at a lower temperature than the standard 37°C since zebrafish are typically housed at 28°C. Fin tissues were not dissociated even after 90 minutes of incubation at 28°C, and cell survival rate was low. We found no difference between incubating tissues at 33°C or 37°C in either uninjured or injured fins (both over 90% survival). Increasing the concentration of Liberase DH (in order to decrease digestion time) led to poor cell survival rate. Additionally, both collagenase IV and Liberase DH were needed for timely dissociation of fin tissue. Lastly, we determined that this enzymatic digestion recipe can be used for both fin and heart tissues. Uninj, uninjured; dpa, days post-amputation; dpi, days post-injury. A (Acceptable) = Over 88% cell survival; Un (Unacceptable)= Not enough cells to check survival; * PBS plus 10mM HEPES, 30 mM taurine, 5.5 mM glucose, and 10 mM BDM (store for 3 months). This recipe was recommended for cardiomyocyte dissociation & culturing from Sander et al., 2013.

Sander, V., Sune, G., Jopling, C., Morera, C., and Izpisua Belmonte, J.C. (2013). Isolation and in vitro culture of primary cardiomyocytes from adult zebrafish hearts. *Nature protocols 8*(4), 800-809.

Mouloungui, E., Zver, T., Roux, C., & Amiot, C. (2018). A protocol to isolate and qualify purified human preantral follicles in cases of acute leukemia, for future clinical applications. *Journal of ovarian research*, *11,* 1-15. https://doi.org/10.1186/s13048-017-0376-6

Yuefeng Li, Kiran Nakka, Thomas Olender, Philippe Gingras-Gelinas, Matthew Man-Kin Wong, Daniel C.L. Robinson, Hina Bandukwala, Carmen G. Palii, Odile Neyret, Marjorie Brand, Alexandre Blais, F. Jeffrey Dilworth. (2021). Chromatin and transcription factor profiling in rare stem cell populations using CUT&Tag. *STAR Protocols*, *2*(3), 100751.

Pimpalwar, N., Czuba, T., Smith, M. L., Nilsson, J., Gidlöf, O., & Smith, J. G. (2020). Methods for isolation and transcriptional profiling of individual cells from the human heart. Heliyon, 6(12).

Adult Caudal Fin Dissociation

BUFFERS

BSA blocked 50 & 15mL conical tubes

HBSS, no Mg^{++}, No Ca^{++}, no FBS

Digestion Buffer: HBSS plus 12.5 µM $CaCl_2$, collagenase IV (5mg/mL) and Liberase DH (0.26U/mL); *Prepare fresh, store on ice*

Stop Buffer: HBSS plus 10% (v/v) FBS +12.5 µM $CaCl_2$

Enzyme	Vendor	Cat#	How to make	Stock	Final	Volume
**Collagenase Type IV**	Thermo Fisher (Gibco)	17104019 (1g)	Add 2mL HBSS to vial	500mg/mL	5mg/mL	5µL per 250µL
**Liberase DH**	Sigma (Roche)	5401054001 (1g)	Add 2mL HBSS to vial	13 U/mL	0.26 U/mL	10µL per 250µL

PROCEDURE

1. Pipette 250µL per 6-10 fins (up to 750µL per tube) of cold **HBSS** in 1.5mL lo-bind microcentrifuge tube (Eppendorf: 022431021). Keep on ice during amputations.
2. Anesthetize fish using 1x tricaine solution
3. Cut either 2DPA or uninjured fish fins and place in the microcentrifuge tube using forceps.
    a. 2-3 fins (uninjured or 2DPA) can be used for ~1 antibodies (unsorted)
    b. ~40,000-60,000 cells per fin
4. Quick spin on the tabletop centrifuge for ~10s
5. Remove HBSS and add 250µL (per 6-10 fins) of freshly made **Digestion buffer**, gently pipette up and down
6. Drop in a 1.5x8mm microcentrifuge stir bar.

7. Set to stir 100-120 rpm at 35°C.
    a. I.e. In a 35°C incubator, place tube(s) on a centrifuge tube rack on top of stir plate set to 120rpm.
    b. Check the temperature of the block or water bath using a thermometer, do not rely on the machine to be accurate.
8. Digest fins for 45-60mins at 35°C
    a. Gently pipette using a p200 every 15-20 minutes to help break up the tissue. There should be no large pieces when finished.
9. During this time, chill the centrifuge and swing bucket rotor (and attachments) to 4°C.
10. Once the sample can be pipetted up and down without clogging, continue to **gently** pipette up and down at room temperature to dissociate any remaining small clumps.
    a. Do not pipette samples more than 3 minutes each.
    b. Be sure to pipette **slowly** and **gently**
11. Add an equivalent amount (750 µL at max.) of **Stop Buffer** and gently pipette up and down. Avoid bubble addition.
    a. Place on ice.
    b. Use pipette tips with large openings
12. Filter through sterile filters of 70 µm size. A larger or smaller filter should be used if collecting for specific cell-types.
    a. Using RO water, wet the entire filter screen. You may see lines where you have presoaked (they appear slightly darker). Make sure you soak the entire filter. Remove any remaining drops of water from the top/bottom of the filter.
    b. Insert cell strainer into a BSA blocked 50mL conical tube pre-filled with 10mL **Stop buffer**.
    c. Add in 1mL **Stop buffer** to coat the filter
    d. Strain cells by placing pipet tip against the filter and ejecting the solution.
    e. Add in additional **Stop buffer** to rinse the strainer. (Usually final between 15-20mL)
    f. Pipette any drops on the outside of the strainer that didn't drop into the conical

tube.

13. Centrifuge at 600x g for 10 min at 4°C to remove digestion enzyme. Remove supernatant, making sure not to disturb the pellet.
    a. Do not try to remove all of the liquid. Consider stopping 0.5mL - 1 mL above the pellet to avoid discarding cells.
    b. The force with which cells are centrifuged changes both cell survival and cell pelleting. Smaller and lighter cells require higher centrifugation to pellet, while larger and heavier cells may be damaged when using this same force. **It may be required to optimize the centrifugation speed based on the desired cells types.**
14. Resuspend with 5mL **HBSS** & transfer to BSA blocked 15mL conical tube
15. Centrifuge at 600xg for 10 min at 4°C
16. Pipet out most of the supernatant, making sure not to disturb the pellet.
    a. Do not try to remove all of the liquid. Consider stopping 0.5mL - 1 mL above the pellet to avoid discarding cells.
17. Resuspend with 2-5mL of **HBSS** (no calcium, no magnesium, no FBS)
    a. If cells prove to be delicate, this second rinse can be skipped.
18. Centrifuge at 600xg for 5 min at 4°C
19. Pipet out most of the supernatant, making sure not to disturb the pellet.
20. Resuspend the cell pellet in 100µL per sample of **HBSS** (no calcium, no magnesium, no FBS)
21. Continue with downstream experiment(s).

Embryo Fin Fold Dissociation

BUFFERS

BSA solution

Egg water, 20x Tricaine

Pronase, 20 mg/mL stock

HBSS, no Mg^{++}, No Ca^{++}, no FBS

Digestion Buffer: HBSS plus 12.5 µM $CaCl_2$, collagenase IV (5mg/mL) and Liberase DH (0.26U/mL); *Prepare fresh, store on ice*

Stop Buffer: HBSS, 10% (v/v) FBS, 12.5 µM $CaCl_2$

Enzyme	Vendor	Cat#	Stock	Final
**Collagenase Type IV**	Thermo Fisher (Gibco)	17104019 (1g)	500mg/mL	5mg/mL
**Liberase DH**	Sigma (Roche)	5401054001 (1g)	13 U/mL	0.26 U/mL

PROCEDURE

Mating

1. The night before mating, sort one male and one female fish into mating tanks using a divider.
    a. Consider using 2 females to one male if many embryos are needed
    b. Wait at least 10 days before trying to mate again.
2. Early the next morning, remove the divider of all tanks at the same time.
    a. Zebrafish prefer to mate in the morning, soon after the light cycle begins.
3. After 1-1.5 hours, collect embryo. The best option is to begin the 1-hour time at first

time mating.

4. Do not continue to collect embryo after this time point.
    a. The embryo must all be fertilized at about the same time to be the same hfp.
5. Move embryo to clean dishes, transfer 50 - 60 embryo per petri dish

Dechorionating

1. At 24hpf, remove dead embryo, and move the live embryo to a 15mL conical tube.
    a. 100 embryos will give a yield of ~75k-100k nuclei.
2. Allow embryo to pellet on their own ~30s.
3. Remove as much egg water as possible.
4. Add in the Pronase Solution + eggwater (2mg/mL working concentration) at room temperature. Incubate for 4 minutes. Invert the tube ~ 1x per minute
    a. **24hpf** should incubate about **4 minutes** before rinsing. Begin removing the Pronase solution at 3m 30s.
    b. If decoronating a different embryonic stage for the first time, use a petri dish instead of 15mL conical tube. Check the condition of the chorions by gently depressing them with tweezers and looking at them under a stereomicroscope. When the chorions no longer return to a spherical shape, the Pronase treatment is complete. Typically, 1-10 minutes, depending on the embryonic stage. Record the age and number of minutes for future experiments.

**Pronase Solution made from solid powder (Roche 165921)**			
	**Stock concentration**	**Working Concentration**	**Volume (mL)**
**Pronase**	20mg/mL	2mg/mL	1.5mL
**Egg Water**			13.5mL

5. Quickly remove as much pronase solution as possible. Add fresh egg water (without

pronase) to the tube. Invert 2-3 times to rinse the embryos in the egg water.

6. Allow embryo to pellet on their own for ~30s. Repeat rinse for a total of two washes.
7. Repeat another 2 washes with HBSS (no calcium, no magnesium).
8. Move the dechorionated embryo to a sterile 60 x 15 mm petri dish (Falcon [Corning]: ref 351007) with a low volume of HBSS and tricaine to anesthetize embryo
9. Most embryo will be dechorionated at this point. The embryo with remaining chorions should easily break open using a pair of tweezers. Gently grab either side of the chorion with each tweezer, and pull apart.

Cutting Fin Fold

10. The easiest way to keep track of the embryo you have cut is to first move all embryo to the center of the dish. (see below figure)
11. Using a scalpel with a curved blade, cut the fin fold of each embryo (Fig 1B). The cut should be made a small distance into the notochord.
12. The fin and/or the embryo may become stuck to the petri dish after being cut. Gently push the stuck embryo off of the dish with the tip of the blade. Move the cut embryo (not the fin fold) to the outer edge of the petri dish (Fig 1D).
13. Cut multiple fin folds.
14. After several fin folds have been cut, remove the cut-embryo from the dish. Be sure not to remove any floating fin folds.
15. To dislodge the fin fold, if stuck to the dish, use the tip of a p200 pipet. Gently using the tip of the pipet, face the opening of the tip so the free-edge of the fin fold will be inside of the opening of the tip (Fig 1E). Gently scrape upwards so the fin fold gets pushed further into the tip (Fig 1F). Do this a few times, gently, until the fin fold is dislodged from the dish.
16. Transfer the fin folds to a lo-bind 1.5mL centrifuge tube (Eppendorf: 022431021) using a

p200 pipet.

17. Move onto fin fold optimized dissociation.

Figure 1

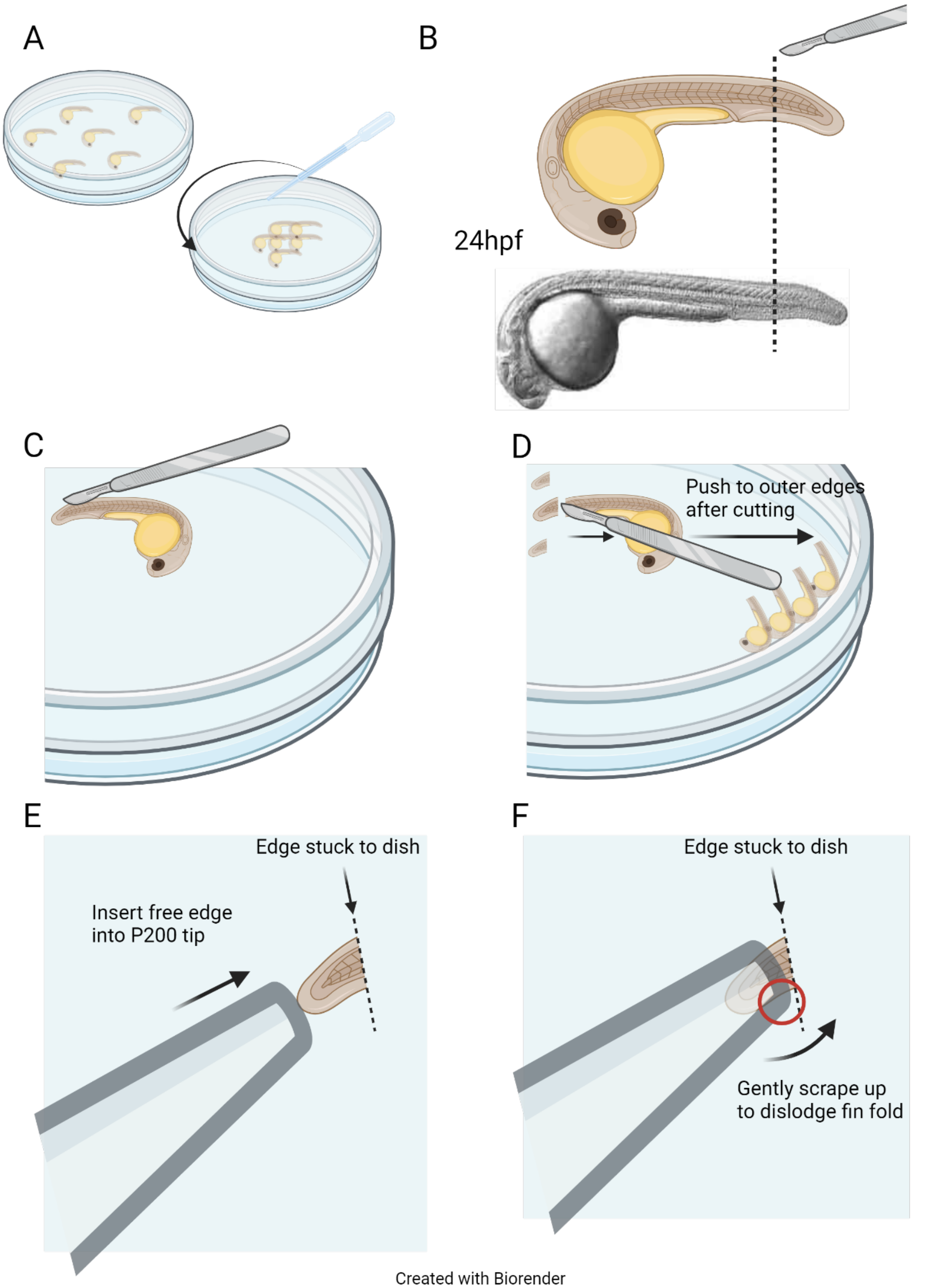

Created with Biorender
24hpf image from ZF-Health the Zebrafish Regulomics for Human Health
http://www.zf-health.org/information/factsheet.html

Fin Fold Dissociation

18. Pre-cool the centrifuge and swing bucket rotor (and attachments) to 12°C.
    a. Embryonic cells are sensitive to cold temperatures. **Do not keep on ice.**
19. Centrifuge fin folds for ~1 min at 300 x g to pellet fin folds (desktop centrifuge is fine for this step)
20. Remove supernatant, add in 250µL Digestion buffer per 100-150 fins folds
21. Place samples in a block heater or water bath set to 35°C .
    a. Check the temperature of the block or water bath using a thermometer, do not rely on the machine to be accurate.
22. Check every 5 minutes, gently pipet up and down with a p200
23. Fin folds should be fully dissociated after 15-20 minutes.
24. Add an equivalent amount (750 µL at max.) of **Stop Buffer** and gently pipette up and down. Avoid bubble addition.
25. Filter through sterile filters of 70 µm size.
    a. Using RO water, wet the entire filter screen. You may see lines where you have presoaked (they appear slightly darker). Make sure you soak the entire filter. Remove any remaining drops of water from the top/bottom of the filter.
    b. Insert cell strainer into a BSA blocked 50mL conical tube pre-filled with 10mL **Stop buffer**.
    c. Add in 1mL **Stop buffer** to coat the filter
    d. Strain cells by placing pipet tip against the filter and ejecting the solution.
    e. Add in additional **Stop buffer** to rinse the strainer. (Usually final between 15-20mL)
    f. Pipette any drops on the outside of the strainer that didn't drop into the conical tube.
26. Using the pre-cooled swing bucket rotor, centrifuge at 600x g for 5 min at 12°C to stop

digestion.

27. Pipet out most of the supernatant, making sure not to disturb the pellet.
    a. Do not try to remove all of the liquid. Consider stopping 0.5mL - 1 mL above the pellet to avoid discarding cells.
28. Resuspend with 2-5mL of **HBSS** (no calcium, no magnesium, no FBS), transfer to BSA coated 15mL conical tube
29. Centrifuge at 600xg for 5 min at 12°C
30. Pipet out most of the supernatant, making sure not to disturb the pellet.
31. Resuspend the cell pellet in 100µL per sample of **HBSS** (no calcium, no magnesium, no FBS)
32. Continue with downstream experiment(s).

Protocol for CUT&Tag

BUFFERS:

Bead Activation Buffer [10mL] (211 uL per sample)

200 µL 1M HEPES-KOH pH 7.9

100 µL 1M KCl

10 µL 1M $CaCl_2$

10 µL 1M $MnCl_2$

9.68 mL ddH_2O

Filter to sterilize; *Store 4°C for 6 months*

Nuclear Extraction Buffer [50mL]

1 mL 1M HEPES-KOH pH 7.9

500 µL 1 M KCl

12.5 µL 2 M spermidine

500 µL 10% (vol/vol) Triton-X100

10 mL glycerol

38 mL ddH_2O

1 Roche Complete Protease Inhibitor EDTA-Free tablet

4°C for 6 months if ***no*** *spermidine or PI is added; 4°C for 1 week if spermidine and PI is added*

Wash150 Buffer [50mL]

1 mL 1 M HEPES pH 7.5

1.5 mL 5 M NaCl

12.5 µL 2 M spermidine

47.5 mL with ddH_2O

1 Roche Complete Protease Inhibitor EDTA-Free tablet

Filter to sterilize; Store 4°C for 1 week

Digitonin (5%)

Dissolve 50 mg digitonin in 1 mL DMSO. Aliquot and store at -20 °C.

Digitonin150 Buffer [500µL]

499 uL Wash150 Buffer

1 uL 5% Digitonin

Prepare fresh, keep at 4°C

Antibody150 Buffer [500µL] (50 uL per sample)

498 uL Digitonin150 Buffer

2 uL 0.5 M EDTA

Prepare fresh, keep at 4°C

Wash300 Buffer [50mL]

1 mL 1 M HEPES pH 7.5

3 mL 5 M NaCl

12.5 µL 2 M spermidine

46 mL with ddH_2O

1 Roche Complete Protease Inhibitor EDTA-Free tablet

Filter to sterilize; Store 4°C for 1 week

Digitonin300 Buffer

499 uL Wash300 Buffer

1 uL 5% Digitonin

Prepare fresh, keep at 4°C

Tagmentation Buffer (300 uL per sample)

297 uL Digitonin 300 Buffer

3 uL 1 M $MgCl_2$

Store 4°C for 1 week

ANTIBODIES

Table 3. CUT&Tag antibody information

Antibody	Host	Company	Catalog #	Dilution
**IgG**	Rabbit	abcam	ab46540	1/100
**Secondary**	Guinea Pig anti-Rabbit	Antibodies online	Abin 101961	1/100
**H3K4me1**	Rabbit	abcam	Ab8895	1/50
**H3K4me3**	Rabbit	abcam	ab8580	1/100
**H3K27ac**	Rabbit	Active Motif	39336	1/50
**H3K27ac**	Rabbit	Active Motif	39133	1/50
**H3K27ac**	Rabbit	Abcam	Ab4729	1/100

- A no-primary antibody control may also be used
- It should be considered to use the same host as the primary antibodies.

PROCEDURE

Nuclei fixation and binding nuclei to activated beads:

1. If you have not already done so, transfer filtered cells to a BSA treated 15mL conical tube, and rinse with 2-5mL HBSS. Centrifuge at 4°C using a swing bucket rotor set to 600xg for 5 minutes. Pipet out most of the supernatant, making sure not to disturb the pellet. Continue to step 3.

2. If cells have already been rinsed and resuspended with 100µL per sample of HBSS, spin cells down at 600xg for 5 min at 4°C. Remove supernatant from cell pellet.
3. Resuspend cells in 100µL/sample cold **Nuclear Extraction Buffer (NEB)**, and pipet gently up and down 3x to resuspend cells. Incubate for 10 min on ice.
    a. If using sorted cells, consider decreasing NEB incubation time to 5 minutes.

4. Spin nuclei down at **750xg** for 10 min at 4°C, remove supernatant from nuclei pellet.
    a. Continue using the swing-bucket rotor and 4°C temperature.
5. Resuspend in 1/2 volume of HBSS relative to NEB volume (so 50µL per sample).
6. While gently vortexing (set to lowest setting) add paraformaldehyde to a final concentration of 0.1% and incubate at room temperature for precisely 2 minutes
    a. Nuclei fixation is preferred since we saw increased nuclear blebbing in unfixed samples.
    b. **DO NOT OVER-FIX**. Fixation over 2 minutes leads to decreased efficiency.
7. While gently vortexing (set to lowest setting), stop cross-linking by addition of 1.25 M glycine to twice the molar concentration of formaldehyde.
    a. I.e. 60µL to every 1mL (so 6µL glycine per 100µL HBSS)
8. Centrifuge fixed nuclei for 10 min **1,200 x g** at 4 °C and remove supernatant from fixed nuclei pellet.
    a. Fixed nuclei appear to be lighter, so you will have to spin faster.
9. Resuspend in 100µL per sample of **Wash 150 buffer** (without digitonin) and transfer to a lo-bind 1.5mL centrifuge tube (Eppendorf: 022431021).
10. Keep on ice until beads are prepped (next section)

ConA bead activation:

11. Gently resuspend the Concanavalin A (ConA) coated magnetic beads and withdraw 11µL/sample (to ensure that there will be 10 µL for each intended reaction). Place the total volume in a single lo-bind 1.5 mL microcentrifuge tube for batch processing.
12. Place the tube on a 1.5 mL magnetic stand and wait 1-2 minutes until slurry clears and pipet to remove supernatant.
13. After removing the tube from the magnetic stand, resuspend beads with 100 µL/sample cold **Bead Activation Buffer**, and mix by gentle pipetting. Place the tube on a magnetic stand until slurry clears and pipet to remove supernatant.

14. Repeat previous step for a total of two washes.
15. Resuspend beads with 11µL/sample cold **Bead Activation Buffer**.
16. Hold beads at room temperature until nuclei are ready.
17. While vortexing gently (set to lowest setting), slowly add the bead slurry to the nuclei. Place on an end-over-end rotator for 15 min at room temperature.

Primary and secondary antibodies binding:

18. Place the tube on magnet stand for 1-2 min until slurry clears, and pipet to remove supernatant.
    a. Keep supernatant to test efficiency of bead binding
19. Resuspend beads in 50µL per sample (N+1) of **Antibody 150 Buffer**.
20. Take 10µL of the bound beads and place into a PCR tube.
    a. Add equal part Trypan blue, and use a hemocytometer to look at bead binding. Do the same with the supernatant from previous step.
    b. There should be very few nuclei in the supernatant and many nuclei bound to the beads. Count nuclei to double check you will have enough per sample
21. Aliquot 50µL per sample into a lo-bind centrifuge tube.
22. Add primary antibody to each tube (typically using 1:50-1:100 dilution, **see Table 3**), and gently vortex.

    Note: For the control IgG, it is recommended to use the IgG antibody generated in the same host species as the experimental primary antibody.
23. Incubate tubes on nutator/rotator at 4°C overnight. Beads should not clump throughout the procedure.

---

24. Quick spin to collect buffer from the lid, and place the tube on a magnet until slurry clears and pipet to remove supernatant.
    a. Note: do not rinse beads after removing primary.
25. Mix the secondary antibody 1:100 in **Digitonin 150 Wash Buffer** and add 100 µL per sample while gently vortexing to allow the solution to dislodge the beads from the sides.
26. Incubate tubes on nutator for 45-60 mins at room temperature.
    a. Flick gently after 30m to prevent the beads from aggregating.

27. Quick spin to collect buffer collected in the lid, and place the tube on a magnet until slurry clears and pipet to remove supernatant.
28. Add 500µL **Digitonin 150 Wash Buffer**. Gently vortex to allow the solution to dislodge most or all of the beads.
    a. Flick to resuspend any remaining beads
    b. DO NOT PIPETTE to resuspend the beads. The beads will get stuck inside of the tip.
29. Repeat rinse. Hold beads in the second wash until Tn5 solution is prepped.

Binding of pAG-Tn5:

30. Mix pA-Tn5 adapter complex in **Digitonin 300 buffer** to a final concentration of **1:20** for 50 µL per sample. (2.5µL Tn5 per 50µL sample)
    a. 1:20 for Tn5 purchased from Epicypher (SKU: 15-1017)
31. After a quick spin, place the tubes on the magnet stand to clear and pull off the liquid from the second rise.
32. Mix in 50 µL of the pA-Tn5 mix while gently vortexing to allow the solution to dislodge most or all of the beads.
33. Place the tubes on a nutator/rotator at room temperature for 1 hr.
    a. Flick the tubes gently after 30m to prevent the beads from aggregating.
34. After a quick spin, place the tubes on a magnet stand to clear and pull off the liquid.
35. Add 500µL **Digitonin 300 buffer**. Gently vortex to allow the solution to dislodge most or all of the beads.
36. Repeat rinse

Targeted chromatin tagmentation:

37. After a quick spin, place the tube on the magnet stand to clear and pull off the liquid.
38. Add 300 µL **Tagmentation buffer** while gently vortexing.
    a. Consider prewarming the tagmentation buffer while the Tn5 incubation is running.
39. Incubate tubes for 1 hr at 37°C for tagmentation.
    a. Check the temperature of the block or water bath using a thermometer, do not

rely on the machine to be accurate.

b. A water bath is the preferred incubation setting.

c. Gently vortex after 30m to resuspend the pelleted beads.

40. To stop tagmentation and solubilize DNA fragments, add 10 µL 0.5M EDTA, 3 µL 10% SDS and 2.5 µL 20 mg/mL Proteinase K to each sample.

    a. Do not remove Tagmentation Buffer from samples before adding

    b. Gently vortex after adding the EDTA before adding the rest

41. Mix by full-speed vortexing ~5s, and incubate 1 hr 55 ºC to digest

    a. Check the temperature of the block or water bath using a thermometer, do not rely on the machine to be accurate.

    b. A water bath is the preferred incubation setting.

    c. Gently vortex after 30m to resuspend the pelleted beads.

    d. A visual cue that your reaction is working is the IgG sample will clump together more than the test samples.

42. Add 300 µL Phenol/Chloroform and mix by full-speed vortexing ~2 s.

    a. Tips: Use relatively new phenol/chloroform (6 months or less). Store at 4°C. Seal with parafilm to prevent evaporation which can change the pH.

43. Now transfer the contents to a freshly labeled phase lock tube (Quantabio: 2302830) and centrifuge at 16,000×g in a benchtop centrifuge for 5 min.

44. Add 300 µL Chloroform and invert ~10x to mix (do not vortex). Centrifuge 5 min room temperature at 16,000 x g.

45. Remove the aqueous layer (top layer) by pipetting to a fresh 1.5 mL tube containing 750 µL 100% ethanol, inverting several times to mix.

    a. It is not recommended to use glycogen to visualize pellet.

46. Place at −20°C overnight to precipitate the tagmented DNA.

    a. Alternatively, samples can be incubated at -80°C for ~2 hours

---

47. Pre-chill centrifuge to 4°C. Centrifuge 30 min at 4 °C 16,000 x g.

    a. A swing bucket rotor is no longer needed. Use a 1.5mL microcentrifuge tube compatible rotor.

48. Carefully pour off the liquid and drain on a paper towel. There is typically no visible pellet.
49. Rinse in 1 mL 100% ethanol and centrifuge 10 min at rt 16,000 x g.
50. Carefully pipette off the liquid. Air dry.
    a. Remove most of the EtOH, then centrifuge again for about 1m. Then using a p20, remove as much of the EtOH as possible.
    b. Always check your pipette tip before discarding the supernatant. Sometimes the DNA pellet can be seen if accidentally removed. If this does happen, eject the EtOH back into the tube and centrifuge again for 2m. Try again with the p20.
51. When the tube is dry (but not overly dry), dissolve in 25µL 10 mM Tris pH 8 or molecular grade water and vortex on full to dissolve. Quick spin & vortex 2x to make sure DNA is completely resuspended.
    a. Optional: incubate the tubes at 50°C for 10 minutes.

Quality control:

- ❖ Take 4µl of the sample, add 0.5µl of any i5 and i7, 5µl of NEBNext High-Fidelity 2X PCR Master Mix (NEB #M0541S), then run for **5 cycles** using the same library PCR protocol as below.

  58°C 5 min
  72°C 5 min
  98°C 30 sec
  5 cycles of
      98 °C 10 sec
      60 °C 10 sec
  72 °C 1 min
  Hold 4 °C

- ❖ Take 5µl of each PCR product, add 5µl of qPCR 2X master mix (qPCRBIO SyGreen Mix; PCR biosystems: PB20.11-01), and run qPCR.
  - ➢ Sample CQ value should be at least 2-3 cycle lower than IgG (or no-primary)

control.

Library preparation and cleanup:

52. For each sample, add 2µL each of barcoded i5 and barcoded i7 primers (10 µM stocks).
53. Add 25µL non-hot start CUTANA High Fidelity 2x PCR Master Mix (EpiCypher) or NEBNext High-Fidelity 2X PCR Master Mix (NEB #M0541S) to each sample and mix.
54. Amplify based on the following PCR setting:

    58°C 5 min

    72°C 5 min

    98°C 30 sec

    12-15 cycles of

    98 °C 10 sec

    60 °C 10 sec

    72 °C 1 min

    Hold 4 °C

DNA cleanup using AMPure XP beads

55. Warm beads to room temperature. Gently swirl to resuspend all beads from the bottom of the bottle/tube. Add **1.3x** AMPure XP beads (Beckman Coulter: A63880) to sample volume.
    a. Ex: 50µL total volume add 65µL AMPure XP beads
56. Pipette to mix. Incubate at room temperature for 5 minutes. Vortex 1s and quick spin to collect solution from lid.
57. Place on magnetic rack for 2 minutes
58. Remove the supernatant, avoiding taking up beads.
59. Without removing the beads from the magnet, add 100µL of 80% ETOH.
60. Wait 30 seconds, then remove all ETOH.
61. Repeat for a second rinse.
62. Remove all ETOH and dry beads for ~2 minutes. Do not over-dry, beads should be a little moist-looking.

    a. Watch carefully, beads over-dry quickly.
63. Add 21µL of 10mM Tris pH 8, gently vortex, incubate 5 minutes. Vortex 1s and quick spin to collect solution from lid.
64. Place on magnetic rack for 2 minutes
65. Remove supernatant to new, clean 1.5mL centrifuge tubes
66. Use 1µL to test concentration using a Qubit

ANTIBODY ASSESSMENT

The accurate method to assess antibody quality is by performing a quality control (QC) analysis to visualize sample quality after library prep. For QC, we are using Agilent High Sensitivity D1000 ScreenTape.

High-quality QC results will show a peak at around 350bp, which is indicative of mononucleosomes. Larger peaks are associated with polynucleosomes. Smaller peaks are either primer/adaptor dimers or the space between nucleosomes (Kaya-Okur et al., 2019).

The next graphs show examples of ideal peak traces for assessing antibodies. We tested three different antibodies against acetylation of the lysine residue at N-terminal position 27 of the histone H3 protein (H3K27ac). Samples were prepped at the same time and with the same cell number. H3K27ac-1 (Active Motif: 39336) showed no signal with traces similar to IgG negative control. H3K27ac-2 (Active Motif: 39133) had a small peak signal around 350bp, however the H3K27ac-3 (abcam: ab4729) trace showed greater signal than Antibody H3K27ac-2. Additionally, we saw more signals at longer, polynucleosome-associated sizes.

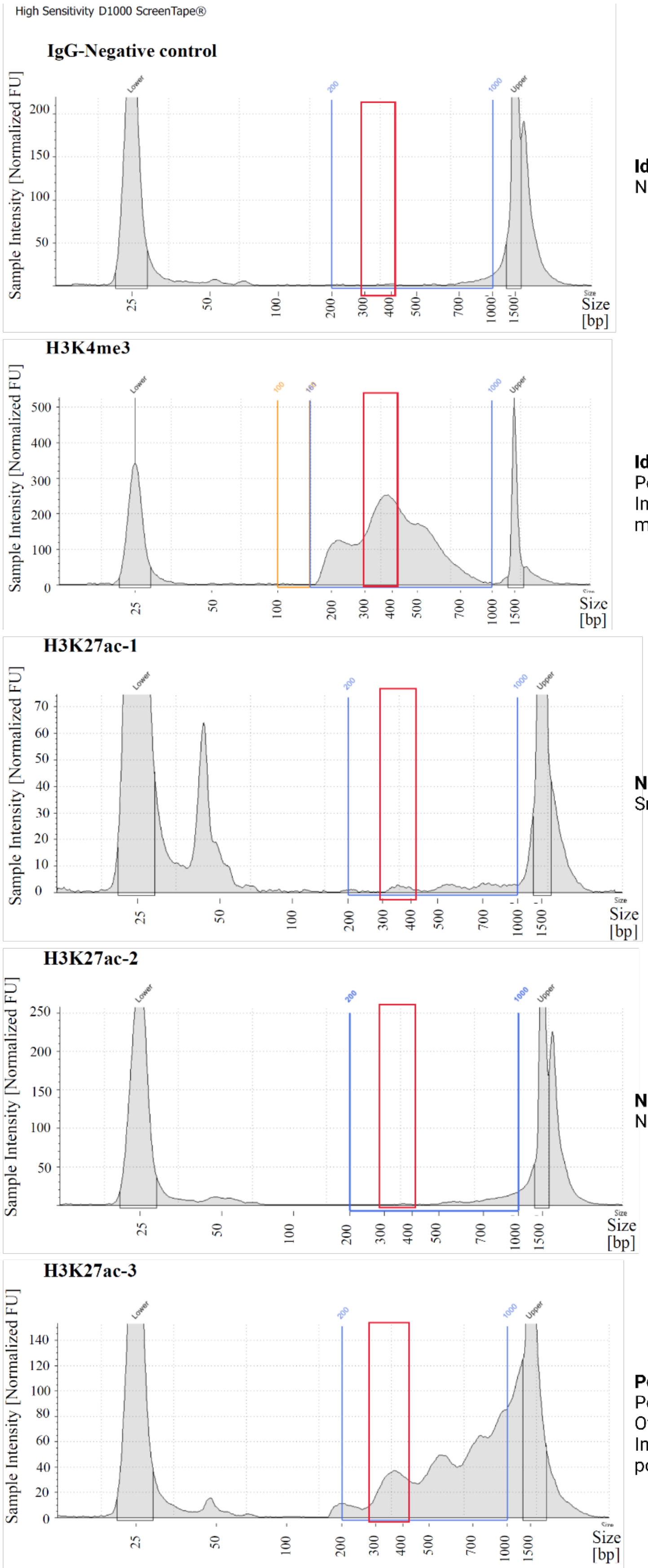

Ideal negative control
No peaks

Ideal sample
Peak at ~350bp
Indicative of mononucleosomes

Not ideal
Small peaks

Negative
No peaks

Positive.
Peak at expected size.
Other peaks present;
Indicative of polynucleosomes.

Made with biorender.com

LIBRARY PRIMER INFORMATION

i5 PCR primer design

i5 Stem	i5 Index	i5 Adaptor
AATGATACGGCGACCACCGAGATCTACAC	Unique 8bp index	TCGTCGGCAGCGTCAGATGTGTAT

Purchased from IDT

Name	Index	Sequence
i5-1	TAGATCGC	AATGATACGGCGACCACCGAGATCTACACTAGATCGCTCGTCGGCAGCGTCAGATGTGTAT
i5-2	CTCTCTAT	AATGATACGGCGACCACCGAGATCTACACCTCTCTATTCGTCGGCAGCGTCAGATGTGTAT
i5-3	TATCCTCT	AATGATACGGCGACCACCGAGATCTACACTATCCTCTTCGTCGGCAGCGTCAGATGTGTAT
i5-4	AGAGTAGA	AATGATACGGCGACCACCGAGATCTACACAGAGTAGATCGTCGGCAGCGTCAGATGTGTAT
i5-5	GTAAGGAG	AATGATACGGCGACCACCGAGATCTACACGTAAGGAGTCGTCGGCAGCGTCAGATGTGTAT
i5-6	ACTGCATA	AATGATACGGCGACCACCGAGATCTACACACTGCATATCGTCGGCAGCGTCAGATGTGTAT
i5-7	AAGGAGTA	AATGATACGGCGACCACCGAGATCTACACAAGGAGTATCGTCGGCAGCGTCAGATGTGTAT
i5-8	CTAAGCCT	AATGATACGGCGACCACCGAGATCTACACCTAAGCCTTCGTCGGCAGCGTCAGATGTGTAT
i5-9	TGGAAATC	AATGATACGGCGACCACCGAGATCTACACTGGAAATCTCGTCGGCAGCGTCAGATGTGTAT
i5-10	AACATGAT	AATGATACGGCGACCACCGAGATCTACACAACATGATTCGTCGGCAGCGTCAGATGTGTAT
i5-11	TGATGAAA	AATGATACGGCGACCACCGAGATCTACACTGATGAAATCGTCGGCAGCGTCAGATGT

		GTAT
i5-12	GTCGGACT	AATGATACGGCGACCACCGAGATCTACACGTCGGACTTCGTCGGCAGCGTCAGATGT GTAT

i7 PCR primer design

I7 Stem	I7 Index	I7 Adaptor
CAAGCAGAAGACGGCATACGAGAT	Unique 8bp index	GTCTCGTGGGCTCGGAGATGTG

Purchased from IDT

Name	Index	Sequence
i7-1	TAAGGCGA	CAAGCAGAAGACGGCATACGAGATTCGCCTTAGTCTCGTGGGCTCGGAGATGTG
i7-2	CGTACTAG	CAAGCAGAAGACGGCATACGAGATCTAGTACGGTCTCGTGGGCTCGGAGATGTG
i7-3	AGGCAGAA	CAAGCAGAAGACGGCATACGAGATTTCTGCCTGTCTCGTGGGCTCGGAGATGTG
i7-4	TCCTGAGC	CAAGCAGAAGACGGCATACGAGATGCTCAGGAGTCTCGTGGGCTCGGAGATGTG
i7-5	GGACTCCT	CAAGCAGAAGACGGCATACGAGATAGGAGTCCGTCTCGTGGGCTCGGAGATGTG
i7-6	TAGGCATG	CAAGCAGAAGACGGCATACGAGATCATGCCTAGTCTCGTGGGCTCGGAGATGTG
i7-7	CTCTCTAC	CAAGCAGAAGACGGCATACGAGATGTAGAGAGGTCTCGTGGGCTCGGAGATGTG
i7-8	CAGAGAGG	CAAGCAGAAGACGGCATACGAGATCCTCTCTGGTCTCGTGGGCTCGGAGATGTG
i7-9	GCTACGCT	CAAGCAGAAGACGGCATACGAGATAGCGTAGCGTCTCGTGGGCTCGGAGATGTG
i7-10	CGAGGCTG	CAAGCAGAAGACGGCATACGAGATCAGCCTCGGTCTCGTGGGCTCGGAGATGTG
i7-11	AAGAGGCA	CAAGCAGAAGACGGCATACGAGATTGCCTCTTGTCTCGTGGGCTCGGAGATGTG
i7-12	GTAGAGGA	CAAGCAGAAGACGGCATACGAGATTCCTCTACGTCTCGTGGGCTCGGAGATGTG
